# Editorial: Functional and molecular insights of neural circuit adaptation, refinement, and remodeling

**DOI:** 10.3389/fncel.2023.1213640

**Published:** 2023-05-19

**Authors:** Alexander Walter, Naofumi Uesaka, Mitsuharu Midorikawa

**Affiliations:** ^1^Molecular and Theoretical Neuroscience, Leibniz-Forschungsinstitut für Molekulare Pharmakologie (FMP), FMP im CharitéCrossOver, Berlin, Germany; ^2^Department of Neuroscience, University of Copenhagen, Copenhagen, Denmark; ^3^Graduate School of Medical and Dental Sciences, Tokyo Medical and Dental University, Tokyo, Japan; ^4^Department of Human Health Sciences, Graduate School of Medicine, Kyoto University, Kyoto, Japan

**Keywords:** synapse, refinement, remodeling, neural circuits, adaptation

Neural circuits are remarkably well-organized systems that transmit and process incoming information. At the same time, neural circuits are highly flexible systems that demonstrate drastic refinement and remodeling depending on animal development, environmental cues, distress, or lesion. Accumulating evidence suggests that precise refinement and remodeling are crucial for appropriate higher brain functions, and disturbance of these processes could account for psychiatric disorders (Luo and O'Leary, 2005; Paus et al., 2008). Refinement and remodeling can be induced by many factors and at multiple scales, but one of the most remarkable changes occurs at the connection within the circuit, i.e., synapses. Synapses are not only hubs that transmit the signals between neurons, but they also adhere two neurons physically in tight proximity. Moreover, synapses are the main computational units for network information processing (Abbott and Regehr, 2004). Therefore, to gain a total view of neural circuit refinement/remodeling and function, we must understand how synapses are assembled and disassembled, how efficient transmission is organized, maintained, stabilized against interference, and adjusted to adapt network processing.

However, the mechanisms underlying the connection and disconnection of synapses, physically and functionally, remain largely unknown. To understand these processes, molecular and functional understanding of synapses, both from the presynaptic and post-synaptic side, including their interactions and structures, are required. Furthermore, it is relevant to understand how disturbances are sensed or cues integrated to adjust to the correct set point. Recent advances in genetic and imaging techniques enable us to address the molecular, functional, and morphological features of synapse refinement/remodeling at multiple levels, such as presynaptic transmitter release mechanisms, post-synaptic receptor properties, synapse adhesion molecules, and synapse/axon growth guidance molecules. It is also becoming possible to analyze the connection and disconnection of individual synapses in a wide range of neural circuits.

A number of reviews have been published in the last decade highlighting the synapse refinement/remodeling (Böhme et al., 2018; Uesaka and Kano, 2018; Wilton et al., 2019; Ibata and Yuzaki, 2021). The scope of this Research Topic is to bring together different researchers studying synapse refinement/remodeling and to compile papers elucidating the molecular and functional mechanisms of neural circuit formation, refinement/remodeling in different approaches such as biochemical, physiological, morphological analyses.

Midorikawa discussed the refinement/remodeling at the presynaptic site, namely the coupling distance between the synaptic vesicle release site and Ca^2+^ channel. The coupling distance is one of the crucial parameters in determining the characteristics of the transmitter release kinetics. The recent technical advances in electrophysiology and imaging are unveiling their developmental- and activity-dependent refinement/remodeling at the CNS synapses. It will be interesting to ask what is shared and what is different between developmental and activity-dependent modulation of the coupling distance.

Two of the studies treat the presynaptic transmitter release properties using electrophysiology and live imaging of Ca^2+^ or glutamate. At the presynaptic sites, several forms of transmitter release have been found, but interpretation and significance of the different forms of transmitter release is still under debate. Grasskamp et al. examined the relationship between the two principal modes of transmitter release: action-potential (AP) evoked and AP-independent, “spontaneous” transmission at the *Drosophila* larval neuromuscular junctions. AP-evoked neurotransmission is considered the primary mode of inter-neuronal communication, whereas spontaneous transmission is required for neuronal development, homeostasis, and plasticity. However, the functional interdependence of both transmission modes remains unknown. The results in the article indicate that the level of spontaneous activity is a predictor of their responsiveness to AP-stimulation. Thus, this seemingly stochastic transmission mode may very well serve as a reference signal to keep connection strength in check and constantly adjust the presynaptic neurotransmitter release machinery. In another study, San Segundo et al. elucidate the significance of the multivesicular release. In the CNS presynaptic terminal, the univesicular and multivesicular synchronous release of neurotransmitter release occurs. They examine the association of glutamate release with excitatory post-synaptic currents by expression of fluorescent glutamate sensor iGluSnFR in the astrocyte which contacts with synapses. Measuring signal of iGluSnFR together with electrophysiological post-synaptic currents at the autaptic hippocampal neuronal cultures, they found that all measured presynaptic terminal possesses a mixed population of univesicular or multivesicular neurotransmitter release and that the terminals showing a preference for multivesicular release display greater strength as well as short-term synaptic depression.

In addition to the functional analysis by electrophysiology or live imaging, the morphological analysis of the synapses under different conditions is crucial to understand the refinement/remodeling of the neural circuit. Feng et al. studied the effects of temperature on the function of the CNS synapses. They show that hypothermia and hyperthermia trigger bidirectional re-organization of presynaptic architecture, synaptic strengthening, and weakening, respectively, at the hippocampal neurons. Interestingly, induction of hypothermia *in vivo* enhances inhibitory synapses in the hippocampus but not in the cortex, suggesting a region-specific form of environmental synaptic plasticity with a mechanism distinct from the classic temperature shock response. Chequer Charan et al. applied cutting-edge serial block-face electron microscopy to study the developmental structural change of the giant presynaptic terminal with multiple active zones, the calyx of Held, located at the medial nucleus of the trapezoid body (MNTB) of the C57BL/6J mouse. By reconstruction of circuitry-level volumes of mouse MNTB at different ages, they found that MNTB neurons reduce in density with age, which is consistent with a type of age-related hearing loss in the mouse. It has been assumed that the mature MNTB neuron is innervated by a single calyx of Held, but they surprisingly observed an average of ~10 % of poly-innervated MNTB neurons along the mouse lifespan.

The aim of this Research Topic was to highlight recent advances in the field of neural circuit adaptation, refinement, and remodeling, both from functional and morphological perspectives. Moreover, we tried to notify the readers of the many open questions that remains to be elucidated to understand neural circuit refinement and remodeling. We hope this Research Topic fulfilled our aim to some extent. We would like to thank all the contributing authors and hope that the readers will share our excitement and enthusiasm to explore the neural circuit refinement and remodeling.

## Author contributions

All authors listed have made a substantial, direct, and intellectual contribution to the work and approved it for publication.
